# PROBLEMS AND DISTRESS AMONG OLDER ADULTS DURING THE COVID-19 PANDEMIC: A LONGITUDINAL THEMATIC ANALYSIS

**DOI:** 10.1093/geroni/igae098.2336

**Published:** 2024-12-31

**Authors:** Carolyn Aldwin, Heidi Igarashi, Maria Kurth, Soyoung Choun

**Affiliations:** Oregon State University, Corvallis, Oregon, United States; Oregon State University, Corvallis, Oregon, United States; The Pennsylvania State University, University Park, Pennsylvania, United States; Oregon State University, Corvallis, Oregon, United States

## Abstract

Despite their greater physical risk and social isolation during the COVID-19 pandemic, older adults reported better mental health and less stress than younger adults, perhaps because they often under-report mental health symptoms and stress in quantitative studies. However, only two small qualitative studies examined stress over time older adults during the pandemic. We examined longitudinal qualitative data on weekly COVID-related problems from internet surveys over an eight-week period early on during the pandemic; 236 out of 249 (95%) respondents reported any problems; of these, 208 (97%) reported problems at three or more time points (nobs=1495, 6.07, SD=2.54, range=3-8). The sample ranged in age from 55-95 (M=71.34, SD=7.29); 74% female, 89% white, and 54% BA+. Using content analysis, we identified 15 codes (Krippendorf’s cu-alpha =.714). Across time, most respondents (76%) described consistent types of difficulties (> 50% of the episodes); problems with everyday protective activities (restrictions, mask wearing) was most common (28%), followed by social connections (missing friends and family; 18%) and psychological distress (17%), often expressed in everyday language, e.g., “cabin fever,” “grinding uncertainty,” or through diacritics (e.g., all caps, multiple exclamation points). However, thematic analysis showed that 27% changed over time, with 7% describing greater social isolation and 9% increasing psychological distress, balanced by 11% who showed increasing adjustment (managing risk). The latter two codes often co-occurred; perhaps navigating an ever-changing COVID landscape increased uncertainty and anxiety. This analysis provided a more nuanced view: problems were near universal in older adults and distress not uncommon.

